# Peliosis Hepatis Presenting As Pseudometastatic Liver Lesions During Adjuvant Therapy for Colon Cancer: A Case Report

**DOI:** 10.7759/cureus.105688

**Published:** 2026-03-23

**Authors:** Fadoua Jebrouni, Hanan Bailal, Asmae Bali, Kaouthar Khater, Hind Chibani, Ouissam Al Jarroudi, Sami Aziz Brahmi, Said Afqir

**Affiliations:** 1 Department of Medical Oncology, Mohammed VI University Hospital, Faculty of Medicine and Pharmacy of Oujda, Mohammed First University of Oujda, Oujda, MAR

**Keywords:** adenocarcinoma, colon, medical, oncology, peliosis hepatis

## Abstract

Peliosis hepatis (PH) is a rare benign vascular liver disorder that may closely mimic hepatic metastases on imaging, posing a significant diagnostic challenge in oncology. We report the case of a 54-year-old patient with left-sided nonmetastatic colon adenocarcinoma (pT4N0) treated surgically and receiving adjuvant Folinic acid, Fluorouracil, and Oxaliplatin chemotherapy for high-risk pathological features. During follow-up, contrast-enhanced computed tomography revealed a dysmorphic liver with a thin-walled lesion in segments II-III, highly suspicious for secondary hepatic involvement in the oncological context. A multidisciplinary decision led to a left hepatic lobectomy, and histopathological examination demonstrated PH, characterized by dilated congestive sinusoids and intraparenchymal hemorrhage, with preserved hepatic architecture and no evidence of malignancy or cirrhosis. Adjuvant chemotherapy was subsequently resumed and completed over a total of six months, with good tolerance. At the last follow-up, the patient showed no clinical, biological, or radiological evidence of disease recurrence. This case emphasizes the importance of including PH in the differential diagnosis of atypical hepatic lesions during oxaliplatin-based chemotherapy to avoid overstaging and unnecessary treatment modification.

## Introduction

Peliosis hepatis (PH) is a rare, nonneoplastic vascular disorder of the liver characterized by the presence of multiple, blood-filled sinusoidal dilatations within the hepatic parenchyma [1]. Its estimated prevalence is approximately 0.13% in autopsy series, highlighting the rarity of this condition [1]. Although often asymptomatic and discovered incidentally, its radiological presentation can pose a significant diagnostic challenge. In patients with a history of solid malignancy, particularly colorectal cancer, the detection of new hepatic lesions during adjuvant chemotherapy is highly suspicious for metastatic disease [2]. However, PH has the potential to radiologically mimic hepatic metastases, creating a substantial diagnostic and therapeutic dilemma. Known associations for this condition include certain medications, underlying malignancies, and chemotherapy agents, which can induce sinusoidal injury. This clinical relevance is heightened in the setting of adjuvant treatment, where accurate staging is paramount for guiding curative-intent therapy. This case report aims to highlight PH as a rare but important differential diagnosis of new hepatic lesions arising during chemotherapy for colorectal cancer, emphasizing the diagnostic pitfalls it may create and the crucial role of histopathological confirmation in guiding appropriate clinical management.

## Case presentation

A 54-year-old patient with no significant past medical history, no known chronic liver disease, no history of alcohol abuse, and no long-term use of hepatotoxic medications was diagnosed with left-sided colon adenocarcinoma.

Clinical examination at diagnosis was unremarkable, with no signs of chronic liver disease. Baseline laboratory investigations, including liver function tests, were within normal limits. The patient underwent surgical treatment on December 19, 2024, consisting of an extended left colectomy, associated with distal splenopancreatectomy, left adrenalectomy, and resection of the perirenal area, due to suspected locoregional extension on preoperative imaging.

Histopathological examination of the surgical specimen revealed a moderately differentiated invasive adenocarcinoma of the colon, infiltrating up to the subserosal layer, with an associated poorly differentiated rhabdoid component. There was no evidence of vascular emboli, perineural invasion, or lymph node involvement. All surgical margins were negative. According to the TNM classification (8th edition, 2017), the tumor was staged as pT4N0.

Given the high-risk pathological features, particularly the T4 stage and poor differentiation, the patient received adjuvant chemotherapy with FOLFOX for three months, consisting of oxaliplatin 85 mg/m² on day 1 every two weeks, leucovorin 400 mg/m², 5-fluorouracil bolus 400 mg/m², followed by continuous infusion of 5-fluorouracil 2,400 mg/m². The treatment was overall well-tolerated.

**Figure 1:**
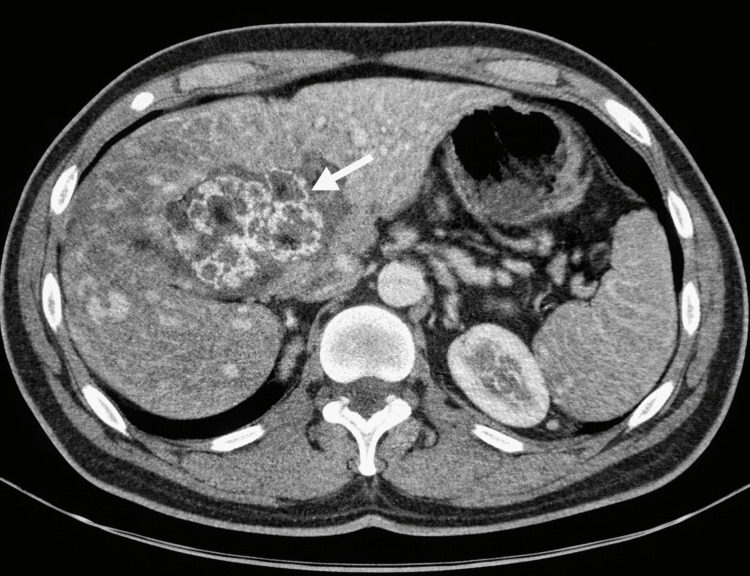
Axial contrast-enhanced abdominal CT showing a thin-walled hypodense lesion in hepatic segments II-III, initially suspected to represent a metastatic disease in a patient with resected colon adenocarcinoma. Final histopathology revealed peliosis hepatis CT: computed tomography

During follow-up, a contrast-enhanced thoraco-abdomino-pelvic CT scan showed no suspicious pulmonary parenchymal lesions, no mediastinal, supraclavicular, or axillary lymphadenopathy, and no pleural or pericardial effusion. At the abdominal level, the liver appeared dysmorphic, with a thin-walled air-fluid collection located in segments II and III, measuring approximately 75 × 40 mm, showing peripheral enhancement after contrast administration, suggestive of secondary hepatic involvement, and associated with extensive locoregional inflammatory infiltration extending toward the transverse colon and the duodenum. There was no intrahepatic or extrahepatic bile duct dilatation, the gallbladder was unremarkable, and no significant abdominal lymphadenopathy was identified (Figure 1).

Given the oncological context and persistent concern for hepatic secondary involvement, the case was discussed in a multidisciplinary tumor board, and a left hepatic lobectomy was performed for diagnostic and therapeutic purposes. The macroscopic examination of the hepatic specimen measured 11 × 5 × 5 cm and weighed 233 g, revealing a 3-mm whitish nodule associated with a ragged area that was extensively sampled.

**Figure 2:**
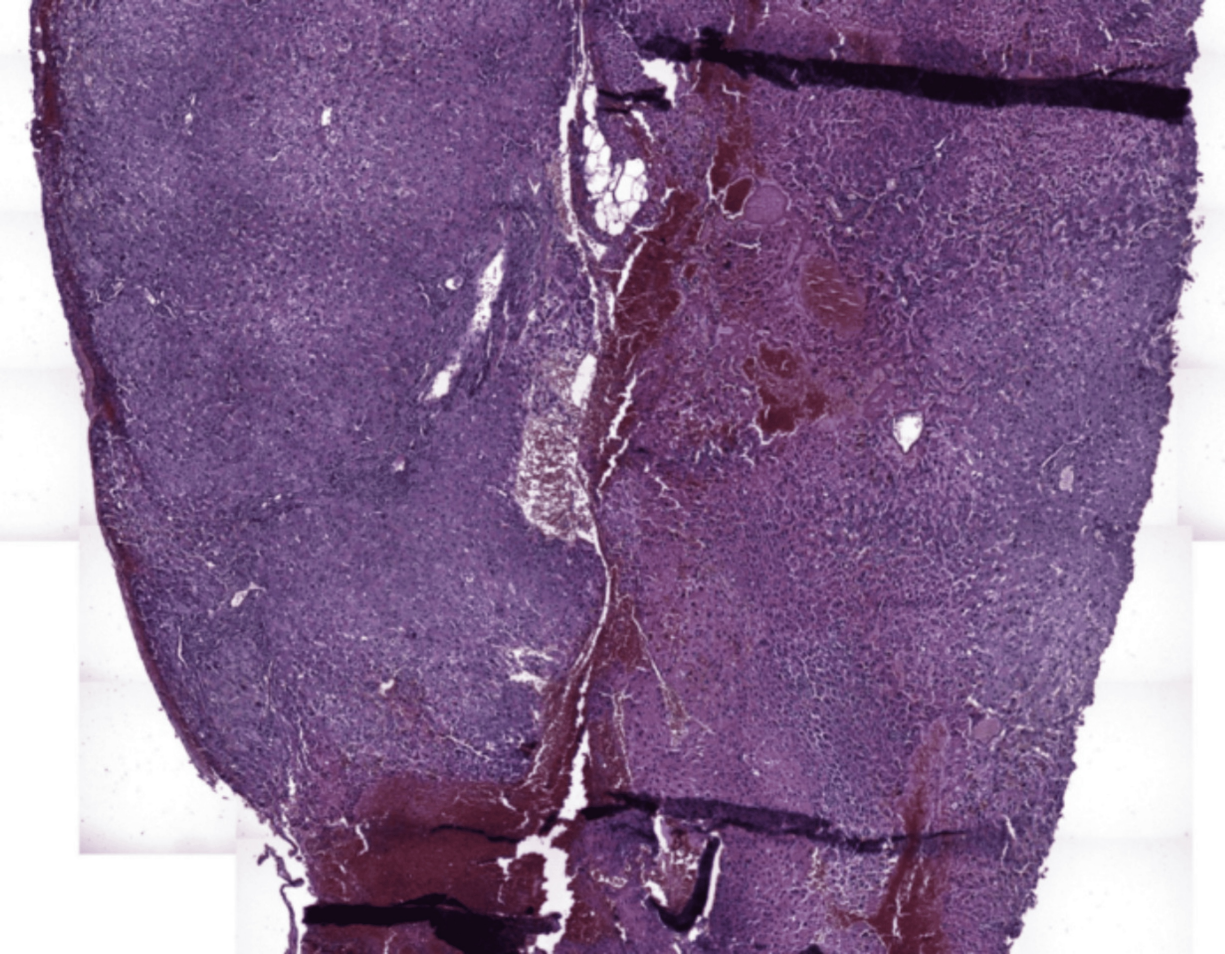
Low-magnification liver biopsy showing peliosis hepatis characterized by irregular, blood-filled spaces without evidence of malignant infiltration in a patient with colon adenocarcinoma (hematoxylin and eosin stain, ×40)

**Figure 3:**
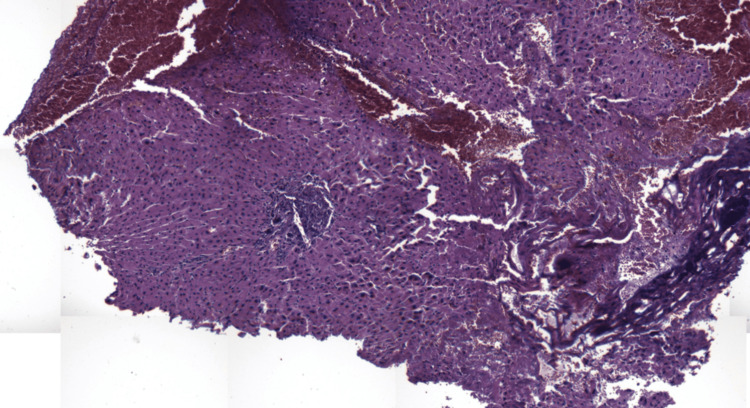
Intermediate-power histological section demonstrating irregular sinusoidal dilatation with blood extravasation and preserved hepatic plates, consistent with peliosis hepatis (hematoxylin and eosin stain, ×100)

Histological examination demonstrated preserved overall hepatic architecture. Portal tracts were not enlarged and contained a moderate polymorphic inflammatory infiltrate, predominantly lymphocytic with rare neutrophils, without interface hepatitis or significant bile duct injury. Hepatic lobules were globally unremarkable. Focally, hepatocytes showed ballooning degeneration with clear cytoplasm, dissociated by areas of intraparenchymal hemorrhage and dilated congestive sinusoids, consistent with PH. Minimal intracellular cholestasis and mild microvesicular steatosis (<5%), predominantly in pericentral areas, were also observed (Figures 2, 3).

There was no evidence of cirrhosis, and no primary or secondary malignant hepatic lesion was identified in any of the examined sections. The final diagnosis was PH-mimicking hepatic metastases in a patient treated for colon adenocarcinoma.

Following histopathological confirmation of PH with no evidence of malignancy involvement, adjuvant chemotherapy with FOLFOX was resumed and completed, for a total duration of six months, with the last cycle (C6D15) administered on October 29, 2025. At the most recent follow-up, the patient remains in good clinical, biological, and radiological control, with no evidence of disease recurrence or progression.

## Discussion

PH is a rare vascular disorder of the liver characterized by the presence of multiple, irregularly shaped, blood-filled cystic cavities within the hepatic parenchyma [3]. Peliosis hepatis is a rare vascular condition of the liver first described in the 19th century [4]. While historically associated with chronic debilitating diseases, infections (such as bacillary peliosis in HIV/AIDS patients), and certain medications like anabolic steroids and oral contraceptives, its occurrence in oncologic patients, particularly those undergoing chemotherapy, has gained increasing recognition [5]. The rarity of PH, especially in a localized form mimicking metastatic disease, underscores the diagnostic challenge it poses in clinical oncology.

The etiology and physiopathology of PH are complex and not fully elucidated, but they are generally understood to involve damage to the sinusoidal endothelial cells, leading to their dilatation and subsequent formation of blood-filled lacunae [3]. In the context of oncologic treatment, chemotherapy-induced sinusoidal injury is a well-documented phenomenon, with oxaliplatin being a prominent causative agent. Oxaliplatin, a platinum-based chemotherapeutic drug widely used in the treatment of colorectal cancer, is known to induce sinusoidal obstruction syndrome, also referred to as veno-occlusive disease [5]. This injury is characterized by damage to the sinusoidal endothelial cells, leading to congestion, hemorrhage, and ultimately fibrosis within the liver. PH can be considered a severe manifestation within the spectrum of oxaliplatin-induced hepatotoxicity, where extensive endothelial damage results in the formation of larger, blood-filled cavities [6]. The mechanism involves direct toxic effects on the sinusoidal lining, impairing the integrity of the vascular architecture and leading to blood extravasation into the space of Disse and subsequent cavity formation.

One of the most significant clinical challenges presented by PH in oncologic patients is its ability to mimic hepatic metastases on imaging studies. The presence of new or growing liver lesions in a patient with a history of colorectal cancer undergoing adjuvant chemotherapy immediately raises suspicion for metastatic recurrence. Imaging modalities such as CT and magnetic resonance imaging (MRI) are crucial for surveillance and diagnosis, but their interpretation can be fraught with pitfalls [7]. On CT, PH lesions may appear as hypodense areas with variable enhancement patterns, sometimes showing peripheral or heterogeneous enhancement that can be indistinguishable from atypical metastases. MRI findings are similarly diverse; PH can present with low signal intensity on T1-weighted images and high signal intensity on T2-weighted images, reflecting the blood content within the lesions. However, the enhancement patterns post-contrast can be highly variable, ranging from early arterial enhancement to delayed, persistent enhancement, further complicating differentiation from hypervascular or hypovascular metastases [5]. The lack of a universally characteristic imaging signature for PH often leads to diagnostic uncertainty. Positron-emission tomography computed tomography (PET-CT) with 18F-fluorodeoxyglucose (FDG) can be a valuable tool in this differential diagnosis. While most malignant lesions, including colorectal metastases, typically demonstrate increased FDG uptake (hypermetabolic), PH lesions are generally isometabolic or show minimal uptake, reflecting their benign vascular nature [3]. This metabolic distinction can be a critical clue, though not always definitive, in guiding further management.

Given the diagnostic ambiguity on imaging, histopathological examination remains the gold standard for a definitive diagnosis of PH. Microscopic evaluation reveals dilated, blood-filled spaces within the liver parenchyma, often lacking a complete endothelial lining or associated with sinusoidal dilatation and congestion [8]. However, obtaining a tissue diagnosis for PH carries inherent risks. Due to the highly vascular nature of these lesions, liver biopsy, whether ultrasound- or CT-guided, is associated with a significant risk of hemorrhage, which can be severe and even life-threatening [3]. Cases of hemorrhagic shock following percutaneous liver biopsy of PH have been reported, highlighting the need for careful consideration of the risk-benefit ratio before proceeding with invasive procedures. In instances where localized PH is indistinguishable from a metastatic tumor and the risk of biopsy is deemed too high, surgical resection may be performed for both diagnostic and therapeutic purposes, as was likely the case in the patient described.

The occurrence of PH in oncology patients, particularly those receiving oxaliplatin, is increasingly recognized in the literature. Several case reports have documented PH-mimicking metastases in patients with colorectal cancer, breast cancer, and other malignancies [9,10]. These reports consistently highlight the diagnostic dilemma and the potential for misdiagnosis, leading to unnecessary changes in treatment plans or aggressive interventions. The presented case adds to this growing body of evidence, emphasizing the importance of considering PH in the differential diagnosis of new liver lesions in patients receiving oxaliplatin-based chemotherapy.

Management and therapeutic implications for PH in oncologic patients are highly dependent on the certainty of diagnosis. Once the malignant nature of the liver lesions has been definitively excluded, often through a combination of advanced imaging (including PET-CT) and, if safely feasible, biopsy or surgical excision, the continuation of chemotherapy can be considered. In many reported cases, PH lesions have shown regression or stabilization after discontinuation or modification of the offending chemotherapeutic agent [11]. However, if the patient requires ongoing systemic treatment for their primary malignancy and the PH is localized and stable, careful monitoring may allow continuation of chemotherapy with appropriate dose adjustments or alternative regimens. The decision to continue or modify treatment must be individualized, weighing the risks of chemotherapy-induced hepatotoxicity against the benefits of oncologic control.

In our case, the patient underwent surgical management of hepatic peliosis, and, given the absence of evidence of malignancy, adjuvant treatment for colon adenocarcinoma was subsequently continued. This case offers several crucial clinical lessons and implications for daily oncologic practice. First, it underscores the importance of a broad differential diagnosis for new liver lesions in cancer patients, especially those receiving chemotherapy known to cause hepatic sinusoidal injury. Overlooking PH can lead to significant overstaging of the disease, potentially classifying a patient as having metastatic (Stage IV) disease when, in fact, they have a benign chemotherapy-induced lesion. This misclassification can result in overtreatment, including unnecessary systemic chemotherapy, all of which carry their own morbidities and impact quality of life. Second, it highlights the value of multidisciplinary discussion involving oncologists, radiologists, pathologists, and surgeons to arrive at an accurate diagnosis and optimal management plan. Finally, it reinforces the need for clinicians to be aware of the imaging characteristics of chemotherapy-induced liver changes and to utilize advanced imaging techniques, such as PET-CT, to aid in differentiation. The ability to distinguish PH from true metastases can spare patients from potentially harmful and ineffective treatments, ensuring that therapeutic decisions are based on an accurate understanding of their disease status.

## Conclusions

In conclusion, this case report serves as a poignant reminder that PH, though rare, is a critical differential diagnosis for hepatic lesions in patients undergoing oxaliplatin-based chemotherapy for colorectal cancer. Its ability to radiologically mimic metastases necessitates a high index of suspicion, careful interpretation of multimodal imaging, and, when appropriate, histopathological confirmations. Recognizing this entity is paramount to preventing misdiagnosis, avoiding overstaging, and ultimately guiding appropriate, patient-centered oncologic management.
